# A framework for synthesis of safety justification for digitally enabled healthcare services

**DOI:** 10.1177/2055207617704271

**Published:** 2017-04-24

**Authors:** George Despotou, Mark Ryan, Theodoros N Arvanitis, Andrew J Rae, Sean White, Tim Kelly, Richard W Jones

**Affiliations:** 1 Institute of Digital Healthcare, WMG, University of Warwick, Coventry, UK; 2 Rotherham NHS Foundation Trust, Rotherham, UK; 3 School of Humanities, Languages and Social Science, Griffith University, Queensland, Australia; 4 NHS Digital, Leeds, UK; 5 Department of Computer Science, University of York, York, UK; 6 School of Engineering, University of Warwick, Coventry, UK

**Keywords:** Health IT safety, safety cases, safety assurance, risk management, SCCI0160

## Abstract

**Background:**

Digitally enabled healthcare services combine socio-technical resources to deliver the required outcomes to patients. Unintended operation of these services may result in adverse effects to the patient. Eliminating avoidable harm requires a systematic way of analysing the causal conditions, identifying opportunities for intervention. Operators of such services may be required to justify, and communicate, their safety. For example, the UK Standardisation Committee for Care Information (SCCI) standards 0129 and 0160 require a safety justification for health IT (superseded versions were known as the Information Standards Board (ISB) 0129 & 0160. Initial as well as current standards are maintained by the NHS Digital.

**Method:**

A framework was designed, and applied as proof of concept, to an IT-supported clinical emergencies (A&E) service. Evaluation was done qualitatively based on the authors’ experience, identifying potential benefits of the approach.

**Results:**

The applied framework encapsulates analysis, and structures the generated information, into a skeleton of an evidence-based case for safety. The framework improved management of the safety activities, assigning ownership to stakeholders (e.g. IT developer), also creating a clear and compelling safety justification.

**Conclusions:**

Application of the framework significantly contributed to systematising an exploratory approach for analysing the service, in addition to existing methods such as reporting. Its application made the causal chain to harm more diaphanous. Constructing a safety case contributed to: (a) identifying potential assurance gaps, (b) planning production of information and evidence, and (c) communication of the justification by graphical unambiguous means.

## Introduction

A patient’s journey through the healthcare system may entail many stages (e.g. preventive, acute, and post-treatment). During all these stages, stakeholders (e.g. doctors, surgeons, general practitioners, nurses, patients), IT systems (e.g. electronic patient records, clinical patient management systems), procedures, drugs and medical devices all collaborate in creating a system implementing patient-centred healthcare services. Furthermore, advances in digital technology have resulted in services incorporating innovative approaches for healthcare. For example, interconnectivity of Electronic Health Records has contributed towards seamless and complete patient records, the use of mobile applications has incorporated the patient more actively into the services, and computer-based clinical decision support systems mine streams of data, offering early warning of potential issues with patients. The resultant services are not randomly assembled, but are designed in order to exhibit certain qualities, one of which is safety. They are dynamically adapting systems demonstrating variability during operation, which stems out of the need for staff to address tensions appearing during operation.^1^ Healthcare services often diverge from intended operation, potentially resulting in harm to patients.^a^ Failure of a service to deliver the intended service may result in conditions which could cause harm through normal operation, known as hazards. Specifically, it has been observed that IT leads to harm through failures, either of the system itself, or by how it was integrated in healthcare operations; this has sparked a discussion on safety of digital healthcare.^2-10^ Although there is growing consensus on the benefits of health IT,^11,12^ there remains the concern of whether we can justifiably have confidence in the operation of such services.

The bigger and more complex a service is, the more it can exacerbate this concern, as there are more opportunities for something to ‘go wrong’. And when it does, the path to harming a patient is often obscure, convoluted and inconspicuous, requiring targeted and systematic approaches to achieving safety. This challenge is not unique to healthcare. Over the years, and motivated by a number of accidents, there has been significant interest on behalf of operators, customers and regulators in being able to capture and communicate the justification of placing assurance on the safe operation of a system. Achieving this requires understanding the conditions during service operation that may result in harm, and justification that they have been avoided or managed in a manner that will result in an acceptably safe service.^b^

In many domains, providers of services are often asked to make a case about the safety of that service, known as a safety or assurance case. This entails a structured argument explaining how the available information allows someone to confidently conclude that a service is sufficiently safe. Safety cases are considered as an effective way of articulating and communicating a compelling safety justification, and are often stipulated as a requirement in many standards and regulatory requirements, including healthcare. In the UK, the safety of clinical IT systems is managed through two standards, complementary to each other: SCCI 0129 and SCCI 0160. SCCI 0160 (*Clinical Risk Management: its Application in the Deployment and Use of Health IT Systems*)^13^ focuses on risk management relating to the deployment and use of health IT in digitally enabled healthcare, whereas SCCI 0129 (*Clinical Risk Management: its Application in the Manufacture of Health IT Systems*)^14^ focuses on the application of risk management to the manufacture of health IT. The standards follow a similar philosophy to that of *ISO 14971 Medical devices – Application of risk management to medical devices*, thereby maintaining a consistent approach to risk management in the healthcare domain. Safety cases have also been considered as a means of contributing towards systematic and proactive management of safety in healthcare.^15^

This paper presents a framework, and its proof-of-concept application, for producing the information necessary for a skeleton safety case about a digitally enabled service. Within its steps, the framework incorporates a deviation-based method for exploratory analysis of a service. The framework described in the paper, can facilitate the operator’s ability to comply with the safety assurance requirements (stipulated in the standard). However, the framework can be applied on its own, as part of the safety assurance process of an organisation.

**Figure 1.**
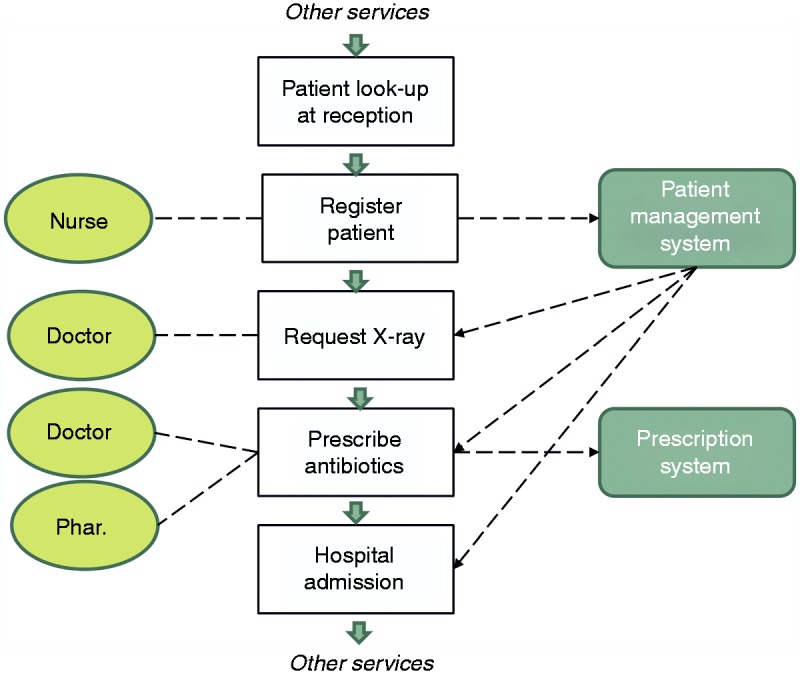
Overview of the A&E service and its health IT dependencies.

The paper uses a case study for proof-of-concept application of the framework, focusing on the use of IT systems in an accidents and emergencies (A&E) clinical service (Figure 1), and motivated by the requirement of SCCI0160 to construct a safety case. It begins with an overview of how understanding potential deviations from intended operation can result in building a causal picture of harm for a service. It follows with explaining the main tenets of justifiably establishing assurance in the safety of a service, as well as how systems analyses can contribute to this. The paper carries on with the definition of a framework which can be applied to a digitally enabled healthcare service, aiming to produce and articulate the safety relevant information and justification. The description of each of the stages of the framework comprises of (a) how it contributes to safety assurance, (b) general guidelines for its application, and (c) proof of concept using the A&E digitally enabled scenario. The purpose of each step is described in the ‘The Safety Justification Framework’ section, whereas guidance, along with example application to the A&E case study, is described in the section ‘Guidance and application of the framework to an A&E services scenario’. Application of the framework would facilitate operators of health IT to comply with the mandatory SCCI 0160 standard, producing the skeleton of a safety justification of the service, a requirement of the standard.

## Understanding the contribution of deviation analysis and safety cases to safety justification

A safety case explains why one should believe that an offered service or system is acceptably safe. Specifically, in the healthcare context, a safety case explains how the available evidence collected during the various phases of the service creation (i.e. design, development and operation) justifies assurance about management of the hazards associated with that service.^16-18^ In the healthcare domain, two standards, mandatory in the UK, specifically stipulate the provision of a safety case for health IT systems, used in clinical services, as a means to document safety assurance.^13,14^ A safety case constitutes the information vessel for the necessary discourse that will provide confidence in the safety of a service.

A number of safety standards in various industries, which aim to represent best practice, are imposed to influence the appropriateness and consistency of the safety-related activities in a domain. Compliance with a standard will result in a suite of information-production activities, assisting with safety justification. Usually, the rigour required by the prescribed processes, in terms of number and depth of activities, reflects the estimated criticality of the system (the more rigorous a followed process is, the safer the system will be). However, prescribing a set of processes relies on that assumption, and does not always explicitly explain how the identified hazards in a system have been dealt with. This has resulted in a number of standards requiring a system to be accompanied by a safety case, which explicitly documents this explanation, based on the information available about a system. The value of creating a structured argument is to have the means of capturing and communicating the rationale about safety, to all involved stakeholders. This systematic approach allows the argument to be reviewed for its completeness and defensibility of claims throughout the project, separating concerns for stakeholders, and revealing any information gaps that may undermine the safety justification.

One of the prerequisites to justifying safety is analysis and understanding of how the constituent elements of a system may contribute to hazards. Among other techniques, the safety analysis process includes a family of methods, described as deviation-based analyses. Such techniques include Hazard and Operability Studies (HAZOP),^19^ Failure Modes and Effects Analysis (FMEA) and Functional Hazard Analysis.^20^ Deviation analyses are exploratory approaches and are used to methodically prompt each part of the system (e.g. patient registration function) with candidate deviations from intended behaviour, usually represented by a guideword (e.g. omission). The analysis then focuses on interpreting the deviation (i.e. patient will not be registered in the system), examining its plausibility and effect, as well as its impact on the operation of the entire system. Deviations considered to be plausible are characterised as potential failure conditions, the effects of which will need to be explored. Although HAZOP is recognised as being prevalent and useful in many domains,^21,22^ the most prominent deviation-based technique used in healthcare has been FMEA. The use of FMEA in healthcare is considered to facilitate a systematic analysis of a service.^23^ FMEA has been recommended by a number of patient safety organisations: the Joint Commission on Accreditation of Healthcare Organizations (JCAHO), as a means for accreditation; the Institute for Healthcare Improvement and the NHS National Patient Safety Agency^24^ as a method in their risk management framework; and the Institute for Safe Medication Practices as a tool for safety analysis. Examples of use of FMEA in healthcare include organ procurement and transplantation,^25^ intravenous drug infusions^26,27^ and communication in emergency care.^28^ Between 2004 and 2008, FMEA was used as a method to improve patient safety as part of the Safer Patients Initiative.^29^ FMEA is seen as an approach to conduct prospective safety analysis in healthcare (most notably as a means of meeting the JCAHO LD.5.2 requirement that asks for proactive analysis). FMEA is considered as a useful technique in proactive safety analysis, particularly in the presence of increasingly complex healthcare services.^26,30,31^ Nevertheless, issues with its reliability are also recognised, particularly variability in results by different teams. However, even in the presence of reliability issues, FMEA is recognised as being effective in engaging the stakeholders in analysis as well as in identifying potential hazards.^32^

Identification of the effects (on safety) of non-intended operation of a service (i.e. failures) will result in understanding the conditions necessary for safety significant events (i.e. risk to the patient). Achievement of safety requires management of these conditions. Risk controls are introduced that will either prevent these conditions, or mitigate their effect (thus breaking the causal chain to harm, or reducing its severity). Justifying the safety of a service (i.e. making a safety case) will eventually entail, among others, arguments appealing to the effectiveness of the introduced risk controls. Stakeholders in charge of the safety case should ideally argue that the end service, along with any introduced controls, will result in acceptable safety.

## The safety justification framework

**Figure 2.**
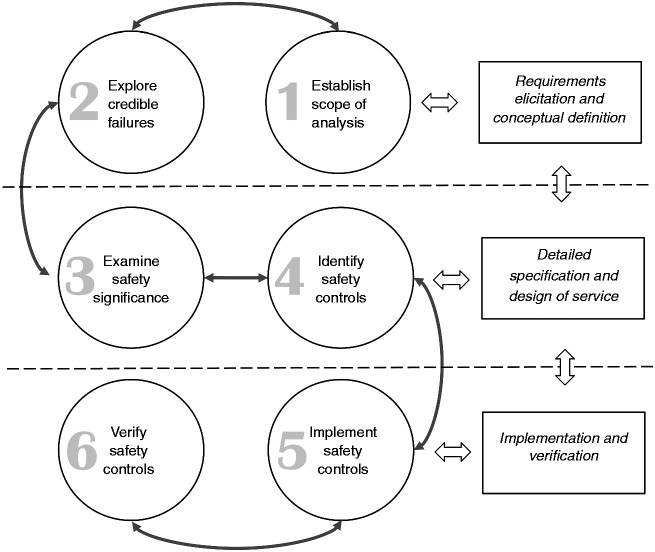
Overview of the framework.

Figure 2 presents the overview of the framework, with its stages categorised according to how they can influence the design of a service^c^ in terms of (a) requirements elicitation and concept definition, (b) detailed specification and design of the system (or service) and (c) implementation and verification of the service. Table 1 summarises the input and output of each step, capturing the information flows during application of the framework.

**Table 1.** Information flow between the framework steps.

	Input	Action	Output
1	Service description documents Related services and dependencies Pathway documents Modelling assumptions and specification	Establish scope of analysis	Adopted service description documents Modelling of gaps between experience and documentation Interfaces of services
2	Models and documentation for the entire service Failure condition guidewords Guidance for specific deviation analysis method used (e.g. clinical FMEA, generic HAZOP etc.)	Identify credible failures	List of credible failure conditions Effects of credible failure conditions Known circumstances leading to failures
3	Credible failures and their effects Existing hazard list or hazard log	Examine safety significance	Relationship of failures to hazards Updated hazards list
4	Updated hazards list List of failure conditions	Identify safety controls	List of safety controls Justification of suitability and effectiveness of each control
5	List of safety controls Justification of suitability and effectiveness of each control	Implement safety controls	Documentation and specification of safety controls List of actions and ownership of safety controls implementation
6	Updated hazards list Justification of suitability and effectiveness of each control Documentation and specification of safety controls	Verify safety controls	Evidence types for each control Assessment of the relevance of existing evidence Plan for generation of evidence

It is not expected that the framework will be used once during the design of the system. Instead, iterative application is expected, and steps of it can be reapplied when new information about the service surfaces (e.g. a new hazard, or a new version of an IT system used) to revalidate the safety justification. The framework can also be applied for appraisal and safety justification of existing services, as is the case of this paper.

### Step 1 – Establish scope of analysis

Step 1 focuses on explicitly establishing the scope of the clinical operation under analysis. It is important during this stage to collect and organise all information available about the operation of the system. Relevant contextual information is necessary in order for the result of the analysis to constitute a valid basis for safety-related reasoning and decisions.^33^ In order for the process to start there should be models of the service under analysis, offering a sufficient degree of representation of its constituent elements (e.g. IT systems, people, procedures). The degree to which a model represents reality is another crucial part of the analysis; if the model is not realistic, then neither will the analysis represent reality, resulting in conclusions that may be irrelevant. It is advised that the analysis team includes domain experts who will review the models. One concern at this stage might be the lack of sufficient or acceptable models. In such a case, a model of the system will need to be created.

The analysis, design and implementation of many systems and services are captured using a generic modelling graphical representation language, such as the Universal Modelling Language (UML),^34^ the Business Process Modelling and Notation (BPMN)^35-38^ or domain-specific languages.^39^ Modelling languages, such as UML and BPMN, are defined in their respective specification documents, which explain their basic elements, and provide the rules on how these elements can be combined. Appropriate use of modelling languages should conform to their respective specification in order to provide a common means of representing a system. In certain cases, the system may be modelled using an ad-hoc language that may not conform to a standard. This is not necessarily a disadvantage if that ad-hoc language is used within an organisation, as it may provide a representation means familiar to an organisation without the need of a learning curve for a new modelling language; it is crucial in this case that the semantics, and any associated notation used, are unambiguous to all stakeholders. However, if the representation is used to communicate with other organisations (e.g. with a health IT manufacturer) an ad-hoc representation may constitute a barrier, and be the source of ambiguity. It is recommended in such cases for an organisation to invest in using a standardised modelling language such as UML or BPMN.

A starting point for creating a model in the health IT domain can be the description of clinical pathways, which documents processes as well as resources needed in delivering care.^d;40,41^ Simply flagging this as an issue to be resolved, without creating clear models of the service, may hinder the ability of the stakeholders to understand potential effects of decisions that will be taken on other ‘neighbouring’ services. For example, the ability of stakeholders to identify the effects of a failure condition to the wider service. Failure conditions at one part of the system may propagate to others, and result in respective (i.e. referring to the latter systems) safety requirements.^42,43^

### Step 2 – Explore credible failures

Step 2 identifies potential deviations from intended operation, which could result in unwanted effects. Deviations always consist of a part of the service (e.g. a task) and a guideword that suggests the deviation with which the service part is prompted (e.g. *‘prescription order’ + ’wrong’*). The guidewords used in these methods provide structure to the analysis, as they guide the analysts through a list of failures commonly affecting safety. This step allows us to identify conditions about the service with a significant effect, thus laying the foundation for building a failure oriented picture. In conjunction with the next step, analysts are then able to understand how the various conditions will contribute to the identified hazards, or identify new ones; also, this offers the opportunity to associate the failure conditions with severity and likelihood, both being constituent attributes of risk, hence transforming the failure conditions picture into a safety picture. Ultimately, it is these failures that stakeholders will manage by specifying safety-related service requirements (describing controls), which will either prevent them, reduce their likelihood of occurrence or reduce the severity of their effect. Once a deviation is considered credible, the analysts will investigate potential causes and the consequences. In addition to the exploratory aspect, analysts have the opportunity to incorporate feedback from reporting as well as tacit experience by discussing known causes.

### Step 3 – Examine safety significance

Step 3 identifies the significance of the effect of the identified failures, by evaluating their contribution to risk. Hazards are conditions of the system which, through normal operation, may result in accidents. It is the hazards in a system that may result in adverse effects to the patient (e.g. injury or death). Safety of a system is achieved by eliminating, or managing, the likelihood and/or severity (risk) of hazards. Achieving this requires understanding of the failures of the system that may constitute the causes of the identified hazards. Risk is the concept that we use to capture the impact of a hazard on safety, and consists of identification of the expected likelihood and severity (of the outcome) of a hazard. Risk assessment of the hazards is necessary in order to understand the contribution of each hazard to overall safety in terms of likelihood and severity.

### Step 4 – Identify safety (risk) controls

Until step 4, the framework focuses on understanding the intended operation of the system, possible and plausible failures, as well as what these failures mean in terms of safety. However, starting with this step the focus moves on to how we can design the system to prevent these failures, or mitigate their effects, as well as how this can be achieved whilst capturing the rationale, which will ultimately result in confidence about both the effectiveness of the controls and their correct implementation. This step prompts understanding of the existing means of managing the hazards, and suggesting new ones if considered necessary. Controls can be introduced at various levels of the operation of the system, and can be technical, procedural, or even organisational (e.g. training and policy), and be owned by both the operator and the manufacturer. For example, risk controls can include: (a) requirements for implementation of a particular safety-related feature in a subsystem (e.g. sanity check of prescribed dose), (b) a requirement that the subsystem will behave in a particular way (e.g. patient list will update every 10 seconds), (c) in-house implementation of a health IT (safety) function, (d) introduction of procedures (e.g. sanity review of drug by nurses before administration), and (e) organisation structure and policy (e.g. periodic training of personnel).

**Figure 3.**
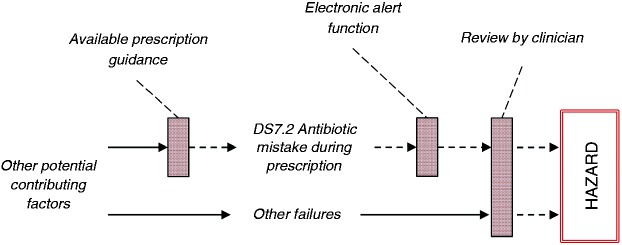
Risk controls introduced to the system to intervene, preventing failures resulting in harm to the patient.

Figure 3 illustrates how the safety controls will provide barriers to the hazard causal chain, preventing a failure from manifesting into one (hazard). The prescription guidance aims to minimise a prescription mistake in the first place, by making sure that clinicians can access guidance when needed. The electronic alert function and the review of the prescription by another clinician attempt to catch the mistake and mitigate its effects. Thus, in this case, all three safety controls will need to not work together in order for a failure to initially present and develop into a hazard. In certain cases, the same safety control will be used for multiple failures; in this case, review of the prescription is a control to both DS7.2 and DS7.6 (see Table 6).

### Step 5 – Implement safety controls

Intention to implement a safety control should not be taken as evidence of its implementation. Step 5 has been designed to reinforce the link between safety-related identified actions and the management process of the service, including management of the implementation of the risk controls. Following identification and justification of appropriate controls, practitioners of the method will need to inquire about their actual implementation. This step focuses on documenting actions, and action ownership that needs to be taken to implement the safety controls. For example, consider the controls illustrated in Figure 3. Implementation of the *review by a clinician* control will involve introduction and documentation of the review in the operating procedures of the organisation, as well as potential training of the affected staff. Implementation of the *electronic alert function* control will involve liaising with the manufacturer of the relevant IT system, to request the required functionality.

Safety controls that will be implemented by the operating organisation are expected to be specified by someone with in-depth knowledge of the healthcare service, whereas the ones that will be implemented as technical solutions may be implemented either in-house or outsourced to a manufacturer. The latter may involve numerous considerations, in addition to the described functionality, that may affect safety. For example, the user interface of the electronic alert function may itself result in harm if not designed appropriately (e.g. a warning pop-up window that can be dismissed accidentally). Other considerations may include the availability of the function (e.g. the IT system offering the electronic alert function being offline); the reliability of the function (e.g. the function not detecting a wrong dosage or dose); functional correctness (e.g. the wrong dosage quantities to be programmed in the function); fault tolerance of the function (e.g. the alert function to be available even when there is a fault with an IT system); and issues such as the development quality of the function (e.g. the testing results of the function). Understanding the safety-related requirements that will need to be met by another organisation (e.g. health IT manufacturer) is crucial to safety justification of a service, and can constitute a safety as well as a business risk.^44^

In order for the operator to be in a position to demonstrate acceptable safety, evidence will need to be provided that substantiates the justification of the appropriateness and correct implementation of the hazard/failure controls. For example, deciding that a *dosage sanity check* control will contribute towards controlling a particular hazard implies intent, and not confirmation that the function exists and operates as required.

### Step 6 – Verify safety controls

Without evidence, a safety justification cannot be confidently supported, but only assumed. Step 6 focuses on the evidence that is required to support the risk controls that have been decided in the previous step. This step should focus on understanding: (a) the essence of what it is that will be claimed in the final justification (e.g. provision of function, a particular operational characteristic such as performance), (b) the types of evidence needed to support the relevant claims, and (c) the explanation of how the available evidence warrants the belief to the claim it supports. The latter is an important part of this step, as often stakeholders, due to their proximity to the operation of the system, may assume logical inferences – not depicted in the safety explanation/argument – which others may not be able to clearly understand. What would be a good combination of evidence to support a claim is not necessarily what is available, or what has been planned to be produced. An inquiry into the sufficiency of available evidence is necessary, to explicitly identify gaps between expectation of support of a claim and availability of information constituting evidence. Upon identification of gaps, it is important for the processes that will remedy the situation to be identified, planned, and assigned to an owner who will see the process through. Internal and external audit to an organisation is a common means to evaluate suitability and sufficiency of collected evidence. Third party audit allows a degree of independence that will evaluate available evidence without the confirmation bias of those who were involved in designing the service. It should be noted though, that (particularly external) audit as an improvement process flourishes in a defined environment, often created by regulation, where feedback is offered and used constructively to improve safety, and not as a blame exercise.

## Guidance and application of the framework to an A&E services scenario

The described framework was applied to a case study for an A&E clinical service, which has allowed for the framework’s evaluation and optimisation. Application of the framework, along with further guidance, is given in the following sections.

### Step 1 – A&E service definition

The basic considerations included by a model should at minimum include: (a) the main activities (e.g. steps of a pathway), (b) the elements or groups of elements that contribute to each step of the service (e.g. systems, subsystems, components, people), (c) the information exchanged between each step of the service and (d) among the elements contributing to each step, as well as (e) the interfaces of the service under analysis with other services.

Table 2 and Figure 1 summarise the elements of the A&E service under analysis. Table 2 presents the main events in the A&E service and the people roles responsible for each event.

**Table 2.** Roles and events in the A&E.

Role	Event
A&E: Reception	Patient arrives with escort Patient registered at reception
A&E: Triage	Patient called through for triage assessment Allergies checked and documented
A&E: Consultant	FBC & X-ray requested and carried out
A&E: Nurse	Dr reviews X-ray report & assesses patient
A&E: Junior doctor	Wound sutured
A&E: Nurse	Refer to on-call team Prescribes antibiotic Refers to the on-take team Patient discharged from A&E
A&E: Doctor	Bed allocated on admissions ward and patient

In this case, the service is modelled in a tabular format by separating aspects of the service (e.g. roles table, activities table). Non-graphical approaches to modelling systems are not uncommon in certain domains. However, it is important to accompany all documents with a definition of the semantics of the words (e.g. action) used, as often the interpretation of the semantics of a word may be different to the aspects that are intended to be modelled. Models created using different modelling approaches (e.g. graphical and text based) can be superimposed as a means of verifying the validity of the models. If one of the two approaches is considered as imprecise, then this may constitute evidence of lack of understanding of the service, whereas if all elements in one model can be traced clearly to the other representation, this is a good indication that the service is well understood, and that the modelling means are well defined. It is advisable that all representations (of the same service) are explored and developed to the extent where all are in agreement.

### Step 2 – A&E deviations definition

The description in this paper presents a generic methodology of applying deviation analysis derived from previous work of the authors;^43^ nevertheless, it should be noted that other well-defined methods can be used instead in this step, such as HAZOP and healthcare FMEA. The output of this part of the method is shown in columns 1–8 of Table 5 (columns 9 and 10 are populated by the next step of the method). Table 5 presents an extract from the deviation analysis method applied to elements of the A&E pathway.

Table 3 suggests a set of guidewords along with their interpretation as a starting point. The proposed set should not preclude assessing the relevance of the guidewords. Practitioners of this method are advised to consider evaluating the suggested guidewords, as well as to assess the suitability of guidewords from other domains. Traditional (stricter) deviation analyses distinguish between guidewords representing deviations of system attributes, from the causes that may result in this deviation. However, a problem with this is that it requires a high degree of familiarity of the practitioners, with the relative merits and limitations of each method, as well as usually requiring a well-defined system model, not always compatible with how clinical services are documented in reality. For the purpose of this method, guidewords should be seen as prompts that will catalyse identification of safety significant failures.^e^ Furthermore, if during application of the guidewords a type of failure is repetitively noticed, but not represented clearly by one of the guidewords (e.g. during application of the guideword other), then analysts may choose to update the guideword list with a new guideword explicitly representing this type of failure. Actively monitoring and updating the set of guidewords will increase confidence in the exhaustiveness of the method, as it will cover both the most representative failures and also explore the applicability of failures from other domains. Finally, it should be noted that over-specifying guidewords may be counter-productive for the method, as it will over-prescribe the expected failures, hindering brainstorming, which is important at this stage.

**Table 3.** Suggested guidewords to begin deviation-based analysis.

Guideword	Interpretation	Guideword	Interpretation
Omission	Something missing when expected	Commission	Something present when not expected
Early	Something happening earlier than expected	Late	Something happening later than expected
Sequence	Something happening out of sequence (when it matters)	Value	Wrong value in a piece of information
Lapse	A person not doing something that they were supposed to	Slip	A person doing something wrong accidentally
Mistake	A person doing something wrong intentionally (unaware that it is wrong – i.e. not malicious)	Access	Someone or something have unintended access to resources or data
More	Unintended increment in the quantity of an attribute of a system element (N.B. needs description of the attribute and its scale)	Less	Unintended decrement in the quantity of an attribute of a system element (N.B. needs description of the attribute and its scale)
Overload	Overloading a system or person (can also be thought of as a specific case of ‘more’)	Other	Generic guideword to encourage free discussion about something going wrong but not covered by the suggested guidewords
Wrong	A generic guideword capturing something wrong happening in the system	Violation	

Once a deviation is considered credible (see Table 5) the analysts will investigate potential causes and its potential consequences. Consequences should be described both in terms of what the deviation ‘means’ for the system element (local *effects*), as well as in terms of how it affects the entire system (*system contribution*). Understanding a deviation from a local viewpoint is always more straightforward than understanding its effects in the entire system, which entails the analysts examining propagation of the effects (and possibly transformation to other types of failures). A clear model, with sufficient resolution of the service, is crucial to establish traceability of the effects of a failure condition to the entire service (Step 1 of the framework). The challenge of understanding service-wide effects is further exacerbated by the dynamic and adaptive nature of clinical services. It is expected that the model of the service will include known variability. Most modelling languages (e.g. UML) provide the constructs to create models capturing a dynamic and adaptive service. Often, a deviation will have already been experienced by stakeholders who may have ‘patched’ the problem by intuitively adjusting their practice. Known circumstances should be recorded when possible, also explaining the source of this explanation (e.g. an incident recording system), as this will then help evaluate the effectiveness of risk controls in step 6 of the framework.

In addition, the deviation table should always contain the source of the system description, as well as the document that captures all rationale generated for each deviation. This should include justification for any deviations that are considered implausible. Finally, it is expected that the team responsible for performing this process may often not have access to sufficient information to determine aspects of a deviation (e.g. its plausibility or effects); the owner of the safety analysis process should take action to gather the required information and ensure that all deviations have been conclusively examined by the end of the entire process.

### Step 3 – A&E deviations credibility assessment and hazard identification

Upon identification of a credible deviation (i.e. failure conditions), its effects need to be understood and, if they are considered safety related, to be associated with a hazard (HazID column in Table 5). Hazards can be identified by (a) previous experience, (b) similar projects, (c) brainstorming and (d) systematic analysis. Although the approach presented in this paper falls within the category of systematic analysis, other sources should not be discounted, and should be used to complete the analysis. The contribution of this method to identifying new hazards is achieved by realising deviations with safety-related consequences that cannot be associated with any of the existing ones. If a failure condition results in a safety-related effect, but cannot be associated with an existing hazard, then introduction of a new hazard may be appropriate. In contrast, if the effects of a failure condition can be related to an existing hazard, then that failure condition is part of the causal chain of that (known) hazard.

An assessment of severity categorisation of each hazard has been included for the purpose of completeness of the example, following the guidance in the SCCI 0160, which recommends a risk framework.

However, guidance on risk assessment is not in the scope of this paper; for this, risk assessment frameworks such as one from the NHS National Patient Safety Agency^24^ can be used. It is expected that organisations will have a local implementation of such risk assessment frameworks. This will also include a process for reconciling different views among the stakeholders performing risk assessment (e.g. independent peer review). It is important that when different standards are used, all stakeholders are clear on what each classification means in operational terms (e.g. what is the interpretation of *probable* likelihood). Possible differences on interpretation should not be left unresolved as they may result in conflicting hazard assessments. Table 4 provides the list of identified hazards ultimately identified using the framework, along with their severity.^f^ If the framework is used as a means to audit an existing service, the process should confirm existing hazards, as well as that all known paths from failures to harm have been managed.

**Table 4.** Identified hazards based on application of the framework.

HazID	Hazard	Severity
H1	Patient not treated	Major
H2	Patient treated with delay	Major
H3	Incorrect patient treatment	Considerable
H4	Introduction of wrong data to patient record	Significant
H5	Unnecessary patient harm/injury during treatment	Considerable
H6	Patient discomfort	Minor

**Table 5.** A&E pathway deviation analysis table.

1	2	3	4	5	6	7	8	9	10	11
FCID	System Element	Type	Document	Guideword	Plausible	Effect	Known Circum/ nces	Contribution	HazID	Notes
DS1.1	Patient registration	Activity	Scenario 2 A&E V1.2.docx	Sequence	Y	Patient not registered in the system	TBD*	Patient not treated or treated with delay	H1, H2	Scenario 2 A&E DevAn Justification.docx §DS1.1
DS1.2	Patient registration	Activity	Scenario 2 A&E V1.2.docx	Omission	Y	Patient not registered in the system	TBD	Patient not treated or treated with delay	H1, H2	Scenario 2 A&E DevAn Justification.docx - §DS1.2
DS1.7	Patient registration	Activity	Scenario 2 A&E V1.2.docx	Value	Y	Wrong patient registered	TBD	Wrong patient record retrieved or new data recorded to the wrong patient record	H3, H4	Scenario 2 A&E DevAn Justification.docx - §DS1.7
DS2.1	Patient triage	Activity	Scenario 2 A&E V1.2.docx	Sequence	N	N/A	N/A	N/A	N/A	Scenario 2 A&E DevAn Justification.docx - §DS2.1
DS2.2	Patient triage	Activity	Scenario 2 A&E V1.2.docx	Omission	Y	Patient not receiving triage may not be treated when critical	TBD	Patient not treated or treated with delay	H1, H2	Scenario 2 A&E DevAn Justification.docx - §DS2.2
DS2.4	Patient triage	Activity	Scenario 2 A&E V1.2.docx	Slip	Y	Wrong triage assessment	TBD	A potential critical condition will be assessed incorrectly	H2	Scenario 2 A&E DevAn Justification.docx - §DS2.4
DS7.1	Prescription of antibiotic	Activity	Scenario 2 A&E V1.2.docx	Sequence	Y	Patient does not receive medication when intended	TBD	Patient will receive treatment with delay and harm may result	H2	Scenario 2 A&E DevAn Justification.docx - §DS7.1
DS7.2	Prescription of antibiotic	Activity	Scenario 2 A&E V1.2.docx	Omission	Y	Patient does not receive medication	TBD	Patient will not receive or will receive treatment with delay	H2	Scenario 2 A&E DevAn Justification.docx - §DS7.2
DS7.6	Prescription of antibiotic	Activity	Scenario 2 A&E V1.2.docx	Mistake	Y	Patient receives incorrect medication	TBD	Patient receives incorrect treatment	H3	Scenario 2 A&E DevAn Justification.docx - §DS7.6
DS8.2	Refer to on-take team	Activity	Scenario 2 A&E V1.2.docx	Omission	TBD	TBD	TBD	TBD	H2	Scenario 2 A&E DevAn Justification.docx - §DS.8.2

* To Be Defined – This implies that a gap in the available information has been identified, which will need expertise and experience, usually from the staff that collaborate for the provision of the service. Ideally, thorough reporting procedures can be used to identify known causes.

**Table 6.** Hazard controls in the A&E pathway.

FCID	HazID	Failure Prevention	Failure Mitigation	Detection	Proposed Evidence	Evidence Sources	Justification	Additional Actions	DSR
DS7.2	H2	Guidance for appropriate prescription at appropriate time	Review of care by other clinician. Electronic prescription Alert based on SNOMED CT diagnosis and prescription record administration - antibiotic has not been prescribed within last x hours	Clinician review/system alert	Training Guidance instruction - Prevention	Training Booklet Source code. System Spec. Testing Scripts and results	Training Guidance should prompt prescription presence check Alert always highlights omission of care to end user Second Clinician prompts review of/consideration of antibiotic prescription	n/a	n/a
DS7.6	H3	Prescription record indicates intended drug and dosage route fields are mandated in order to complete prescription. Prescription fields prompt an entry in each column electronic guardrails prevent dosage outside of therapeutic range. Check against the antibiotic prescription guidelines from organisation	Nurse checks prescription for completion. Clinician review for appropriateness of antibiotic. Pharmacist reviews on-going prescription	Nurse checks prescription for completion. Clinician review for appropriateness of antibiotic. Pharmacist reviews on-going prescription. Electronic Check of dosing and allergies	Nurse checks prescription for completion. Clinician review for appropriateness of antibiotic. Pharmacist reviews on-going prescription. Electronic Check of dosing and allergies	Audit data. Testing Results	Testing results show correct specification and function of system Pharmacy data identifies breakthrough	n/a	n/a

### Step 4 – A&E safety controls

Table 6 provides an extract of the analysis summarising the controls related to the contribution of failures DS7.2 and DS7.6 to hazards H2 and H3. To help with brainstorming and elicitation of the failure controls, the table provides three categories of safety controls, common to safety engineering, which are prevention, mitigation and detection of the failure. The prescription guidance aims to minimise a prescription mistake in the first place, by making sure that clinicians can access guidance when needed. The electronic alert function and the review of the prescription by another clinician attempt to catch the mistake and mitigate its effects. In this case, all three risk controls will need to not work in order for a failure to develop into a hazard. In certain cases, the same safety control may be used for multiple failures; for example, in this case review of the prescription is a control to both DS7.2 and DS7.6.

At this stage, the stakeholders of the analysis can start thinking about the exact way in which the controls will operate, and specify the details of these functions in the service; for example, how the prescription guidance will be provided, whether clinicians will be trained to use it, and whether they will have sufficient time to use this control appropriately. All these concerns will then be verified in the following steps of the method, by looking for data confirming that clinicians actually use the guidance, or any counter-evidence that they are not, such as reports from staff. If data collected during verification is not sufficient to warrant the veracity of the claims about the safety controls, then the safety analysts may plan to collect more data, or redesign the controls in a more suitable way. This kind of interaction is an example of how the framework will facilitate and guide the safety evidence discovery process.

**Figure 4.**
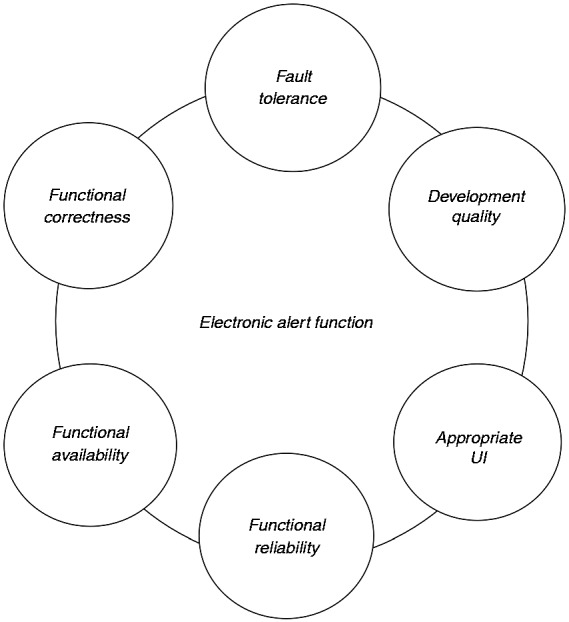
Safety analysis will reveal requirements for health IT functions, from numerous points of view.

Safety controls that will be implemented by the operating organisation will be specified by the clinicians, whereas the ones that will be implemented by the manufacturer of IT (or other systems) may be implemented either in-house or outsourced to a manufacturer. Figure 4 shows an overview of the issues of an IT-implemented function that may be influenced by the safety analysis. Understanding the safety-related requirements that will be discharged to another organisation (e.g. health IT manufacturer) is crucial to safety justification of a service, and can constitute a safety as well as a business risk.^44^

### Step 5 – Tracking A&E safety control implementation

**Figure 5.**
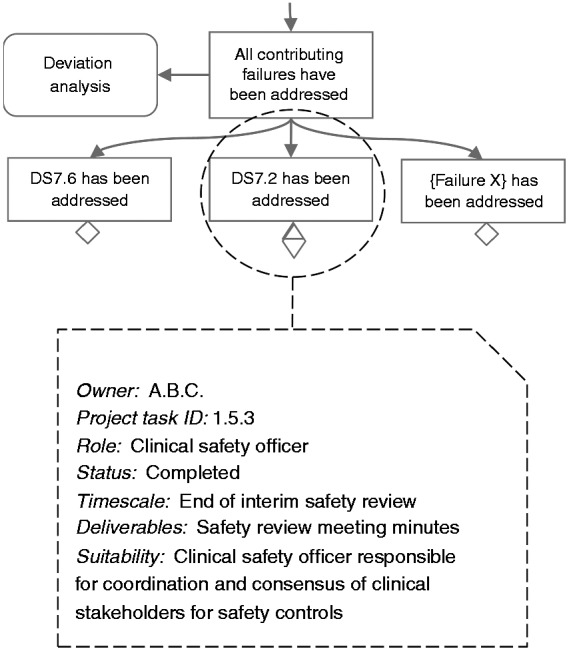
Ownership allocation, and management information annotation, of safety justification components.

Implementation of the safety controls should be tracked and confirmed. This can be done using ‘traditional’ project management techniques such task lists and task tables. The task should contain the necessary information for the person in charge of safety to be able to check progress at any point. This will contribute towards checking for potential delays either due to resource allocation or due to difficulty producing the necessary information. If the latter is the case, this may constitute a risk for the safety justification of the service. For example, if a health IT subcontractor cannot produce evidence for the required behaviour of the medicine administration function, which is a safety control managing a risk, then alternative means of justification should be considered. Even if the function is implemented, not being in a position to convincingly argue about the required behaviour will result in a valid but unsubstantiated or unconvincing justification. Figure 5 illustrates an example of how the annotations on the graphical representation of the argument, (see the ‘Visualising the justification synthesis’ section of the paper), can be used to track information about implementation. In this case, the annotations inform that the clinical safety officer, who is the coordinator of the overall safety case, is also responsible for identifying how failure DS7.2 can been addressed, which involves identification of suitable safety controls. This is something that would ideally involve multiple stakeholders, depending on the type of the control, hence the owner of this claim will need to produce the decisions from the relevant meetings. In this example, the clinical safety officer is the most suitable role for this responsibility,^g^ as according to SCCI 0160 that stipulates the role, they are tasked with this kind of coordination.

### Step 6 – A&E safety controls evidence collation

An extract of the evidence used for the A&E scenario, namely evidence for controls relating to DS7.2 and DS7.5, is shown in Table 6. For example, evidence for the implementation of the review process can be found in the documentation of the operating procedures, demonstrating that the process has actually been introduced. For IT-based controls such as the electronic alert function, suitable evidence may be the documentation of the function, as well as references to the quality assurance of the manufacturer who developed the function. When identifying evidence, its source should also be defined, such as testing reports, system specifications and other studies. In this case, the identified evidence can be found in the training booklet, the system specification, and the testing scripts and results of the prescription system. As with the rest of the safety case, annotations indicate the person, role or team responsible for their maintenance and update.

## Visualising the justification synthesis

**Figure 6.**
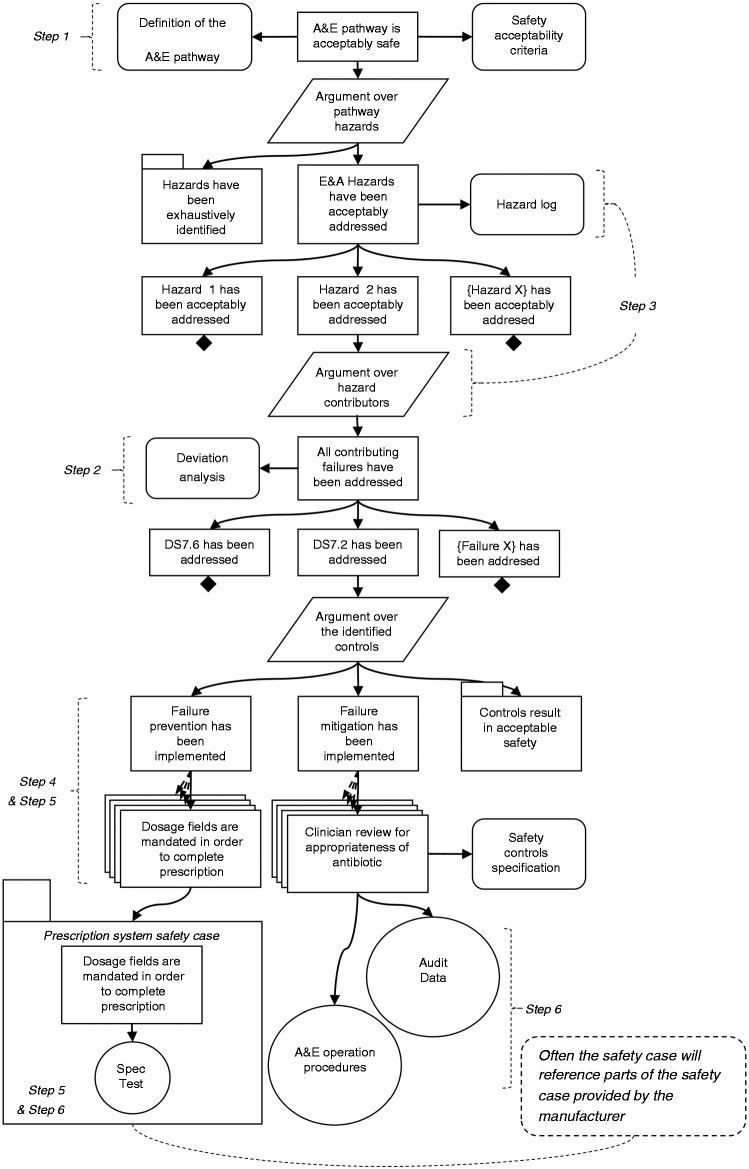
Graphical Representation of the Hazard Argument using GSN (notation assumptions: rectangles: claims, rounded rectangles: contextual information, parallelograms: strategies, circles: evidence, rectangle with smaller rectangle on top left denotes a separate argument (module), diamond: claim to be supported, arrows: inferences).

The reasoning behind the claims in a safety case can often be complex, reflecting the complexity of the service itself. Figure 6 visualises the justification that argues identification and management of the failure conditions that have been identified as potential causes of hazards.

Use of free text to capture the argument often results in arguments that are difficult to follow, thus making the case (more) unclear and incomprehensible. It is believed that supporting text by capturing parts of the safety case in a graphical format contributes towards the clarity of the safety case. The Goal Structuring Notation (GSN) is a language containing all the necessary concepts to capture an argument in a structured format. GSN comes with a graphical notation and a method that can be used to facilitate the construction of an argument.

Figure 6^h^ illustrates a basic argument explaining how the management of the identified hazards allows assurance for the acceptable safety of an A&E service, using the GSN. The top-level claim of the argument, which also constitutes the overall position that we would like to communicate, is about the acceptable safety of the A&E service. This claim is also associated with two placeholders pointing to contextual information about how the A&E service is designed, as well as any safety targets for the service that may be applicable, either qualitative (e.g. introducing controls for all hazards) or quantitative (e.g. previous or desired risk levels). The strategy that follows explains that the claim will be supported by making an argument about managing the identified hazards, stated in the following claim (*A&E hazards have been acceptably addressed*). That last claim is stated in the context of a reference to the hazard log that will provide the source of the hazards for the service. At this point the argument also makes a reference to an argument module (i.e. a self-contained argument), which will explain why we believe that the hazard log is complete; however, this is outside the scope of the actual hazard management argument (and outside the scope of this paper) and thus has been packaged separately.^i^ A claim about addressing each one of the identified hazards is made. A strategy below the claim about addressing Hazard 2 explains that the argument will be further developed by addressing the contributing failures to the hazard, which have been identified during the second and third stages of the framework (i.e. deviation analysis).

## Results

In terms of provision of the clinical service, the framework has provided a valuable means of structuring the information collection processes. It offers interfaces with management of the various tasks, the input of which is necessary for the safety case. Creating a structured safety justification resulted in understanding of what the safety analyses need to provide in terms of information in order to create a convincing argument, and facilitated planning of these activities. Creation of a graphically supported safety case to articulate the argument allowed for clear communication of the overarching safety strategy of the organisation.

The inherent thoroughness of the deviation-based analysis was beneficial towards confidence that all operational aspects have been considered. The exercise resulted in identification of a set hazards that was in accordance with the hazards expected to be found in a real A&E service. There was additional value provided by the framework, of mapping how the various day-to-day failures can result in these hazards, and enabling a systematic review and justification (backed by evidence) of safety controls. The exercise allowed flagging of the information that needed to be provided by representatives from each role (e.g. doctors, nurses, IT contractors) contributing to the service, eliciting tacit expertise that would otherwise have remained concealed, or would have been replaced by assumptions.^j^ The steps of the framework identified numerous dependencies between the safety justification process (and the safety officer responsible for it) and clinical as well ICT staff within the organisation. Clinical and ICT staff dependencies included contribution to the analyses based on their expertise, as well as provision of information that can be used to understand the service, or as evidence to support the safety justification. Finally, application of the framework resulted in clear allocation of ownership of the elements that constitute the justification (e.g. safety controls, deviation analysis, evidence) to specific stakeholders such as the clinical officer, ICT staff and ward directors, thus documenting the responsibility of each stakeholder group towards safety (e.g. provision of IT function).

## Discussion and conclusions

The application of the framework to the A&E scenario was evaluated based on the authors’ expert opinion, from the perspective of a service owner, a regulator and an auditor.^k^ From a clinical operator’s point of view, constructing a safety case allows the explicit reference of all risk controls in place, along with any procedures and evidence of their operation. From a regulator’s and auditor’s viewpoint, application of the framework allows clear association of safety controls and hazards for which they are intended, highlighting the rationale, the specification of the controls, and evidence for their implementation. One downside of using a graphical notation is the resources needed to train personnel to use it, although this is considered to be few by comparison with the entire organisation’s resources for safety. Deviation analyses are inherently subjective, as the system stakeholders interpret the failure communicated by a deviation, as well as its effect. Depending on the experience of the stakeholders, they may capture divergent interpretations, which would need to be resolved and agreed. The obvious downside to this is the increased resources that are needed to disambiguate the effect of the deviations. However, this can also be seen as a strength of the approach, as this kind of discrepancies of expectations among stakeholders may undermine confidence in the safety of the service, if undetected. The deviation analysis approach is very suitable to understanding the causal chains to harm, from unintended operation, in proactive analysis. Other techniques may also be applicable, which can provide the same information flow, as suggested by Table.1. The framework has been particularly useful to highlight the dependencies of information, necessary for the safety justification, between ICT staff, clinical staff and the clinical safety officer, responsible for the overall coordination of the safety activities. Furthermore, the framework identified dependencies of the operator organisation on the manufacturer, which may become areas of business risk if not addressed at the appropriate time. Applicability of the framework goes beyond the scope of IT-based healthcare services, and it can be applied to paper-based systems. In this case, functions performed by IT, such as storage and processing, are performed by other elements of the system (e.g. paper-based archives and person-based processing). Nevertheless, the increasing use of health IT in healthcare has contributed to the complexity of services. IT also affects the way safety can be achieved; risk controls can be introduced as IT functions, technical IT failures can potentially cause harm to patients, and the continuous evolution of IT technologies offers little time for an in-depth understanding of the technologies, requiring the operational and clinical stakeholders to be able to work closer and in a structured and effective way with the technical stakeholders. Although many of the safety-related challenges already existed, health IT has exacerbated the need for a systematic, proactive analysis. This need is also seen by the increasing attention on safety of health IT, such as the SCCI standards and their requirements for a safety case, which was the motivation for this work.

Although the approach has been considered very useful for eliciting failures, risk controls, and establishing justifications, there are certain limitations. Application of the framework needs clear ownership; a stakeholder who is responsible for the completeness and correctness of the process, with the necessary authority to manage the relevant activities described in the framework. Application of the framework in the context of the SCCI standard 0129 and 0160 would not be challenged by this, as these standards stipulate the role of the clinical safety office having the ownership of the process. Although it is expected that most organisations will have a distinct safety role, this should not be taken as granted. Another limitation (and also a common misconception about safety cases) is that application of framework will not necessarily make the service justifiably safe. It provides the necessary information and structure for a proactive analysis, but ultimately, safety assurance will depend upon correct application of the steps, acting upon the findings, and making a convincing argument.

The framework can be seen as a generic and systematic approach to generating information that, in the authors’ view, should constitute the minimum expected to be found in safety justification of any service. Application of the framework performed strongly in enabling a clear understanding of how every piece of information produced contributed to the justification of the service; for example, how evidence about the correctness of an IT function offered by the manufacturer allows the operator (healthcare organisation) to have confidence in the safe operation of the IT system, and in extension the entire service. This is achieved by understanding the relationships between information, and how they are all assembled into one coherent argument about the entire service. Nevertheless, a dynamic system such as a healthcare service is expected to undergo numerous changes, which may undermine the relevance of the identified conditions, as well as the resultant justification (safety case). It is important for the owner of a safety case to establish the processes for continuous monitoring, and update the produced information and justification, according to changes. Updating should be approached with the same systematic manner, following the traceability offered by the framework.

The framework used in the case study was designed for use in the healthcare domain, based on the experience of the authors. It combines a number of techniques and methods used in safety in a way that is considered intuitive for potential users. The framework underwent adjustments based on the findings of the A&E scenario in order to be optimised for healthcare. Although all steps are expected to be part of safety analyses processes in all domains, they are often stipulated as part of regulation or an applicable standard. In healthcare, such standards are not that well known, resulting in the need for some aspects of the safety analysis to be built in the framework, as suggested by this paper. For example, step 1, that considers the definition of the service, was added to the framework, as the authors have experienced a lack of structured and clear description (i.e. models) to be a common barrier for structured safety analysis in healthcare. In other domains (such as aerospace and the process industry) definition of the service is guided by other associated standards, and is not always an integral part of the actual safety analysis process.

The authors believe, based on their experience, that the framework is applicable to any healthcare service, or clinical setting. The framework was designed based on the information necessary to establish any safety case, rather than on the needs of specific case study. The A&E service was seen as a complex, socio-technical system, consisting of numerous elements collaborating in an intended manner to offer the required functionality. It includes generalised (safety) principles that have been used repeatedly in multiple domains. However, evaluation of the degree of generalisation of the framework was considered as beyond the scope of this work, and given the limitation of applying the framework only on the A&E case study, this claim should be taken as a firm belief of the authors rather than as a conclusion of this work.

Concluding, the authors believe that applying the process described in this paper will provide a useful foundation for a concrete and proactive safety discussion, analysis and justification process.

## Notes

a. Harm in the context of this paper implies risks to which patients are exposed due to the operation of the service they use, and not the inherent risk of a disease. This may, for example, include harm caused by the system, or inefficient treatment. The terms adverse event, e-iatrogenesis, sentinel event, safety incident, accident are considered synonymous to harm, which is interpreted as compromise of the safety of a patient.

b. The authors use the terms system and service interchangeably in this paper, implying that a service is an intended operation of a designed assembly (i.e. system).

c. This classification can be applied to both new and existing services; for the latter it takes the function of appraisal and improvement of the intended outcomes.

d. Attention should be paid when using pathway documents as there is a significant variation between organisations on how pathways are document and therefore variable suitability as a source.

e. More about the pedigree of the deviation-based part of the method in Despotou.^43^

f. Likelihood of the hazards was not addressed in the exercise, as this exercise focused on verification of a hazard list based on experience, and identification of the conditions that lead to them. Likelihood assessment would need input from quantitative data (i.e. reporting) or expert opinion (e.g. “in my opinion I see this failure a few times a week”), not available to this proof-of-concept application.

g. The authors have adopted the term clinical safety officer as suggested in SCCI 0160. Although the specific term may not be used in all organisations, it is expected that all organisations performing even rudimentary safety analysis will have a role that will fit the description of a clinical safety officer as stipulated in SCCI 0160.

h. A graphical representation is one of the ways a safety case can be captured. Regardless of the means of capturing a safety case the concepts remain the same. The authors have applied ‘loosely’ the GSN notation, as explanation of the notation is outside the scope of the paper. The GSN standard, which fully defines the notation can be found at http://www.goalstructuringnotation.info.

i. It is expected that a more comprehensive safety case would include elaboration of this argument.

j. A number of TBDs in the tables indicate the need to unearth more expert knowledge. In a large-scale case study of the actual service, identification of an information vacuum would have raised a task to consult the relevant experts within the organisation, or activities, aiming to generate the missing information.

k. The authors collectively have expertise in all of these roles.
